# Awareness About Total Knee Arthroplasty Among Hail Population

**DOI:** 10.7759/cureus.33260

**Published:** 2023-01-02

**Authors:** Ahmed O Alshammari, Nasser A Altamimi, Faisal F Alshammari, Ohud A Altamimi, Abdullah M Aljarboa, Abdullah S Alshebli, Jamal A Almarshadi, Fahad H Alshammari, Adel H Alhammad, Hutaf N Binayesh, Khaled H Almuhaihi

**Affiliations:** 1 Medicine and Surgery, University of Hail College of Medicine, Hail, SAU; 2 Orthopedic Surgery, King Fahad Medical City, Riyadh, SAU; 3 Emergency Department, King Khalid Hospital, Hail, SAU

**Keywords:** knee osteoarthritis/koa, population, hail, total knee replacement, awareness

## Abstract

Background: Knee joint osteoarthritis is the most common among older adults. The knee joint needs to be treated surgically. The prevalence of this disorder is believed to be higher than 40% among people over 60.

Objective: To study public awareness about total knee replacement among the Hail population and to explore the relationship between the level of awareness and different socio-demographic factors.

Methods: A cross-sectional study was carried out in Hail, Saudi Arabia, using an online self-administered questionnaire created via Google Forms. Collected data were coded, entered, and analyzed using the Statistical Package for Social Sciences (SPSS; Version 23).

Results: A total of 431 participants enrolled in the study, of which 242 (56.15%) were males and 189 (43.9%) were females. Out of a total of 34 points, the average awareness score was 15 ± 6 (range: 5-33), with a mean percentage of awareness of 44.1%. The most reported causes of total knee replacement were overweight or obesity (82.6%), aging (61%), and rheumatoid arthritis (45.2%). The most known symptoms were pain (85.6%) and clicking sounds during movements (64.7%). Regarding the knowledge about the treatment, both physiotherapy and weight reduction were mentioned by 290 (67.3%), exercise by 244 (56.6%), and surgical intervention by 177 (41.1%) participants. The purpose of knee replacement surgery was to relieve pain according to 357 (82.8%) participants, improve walking quality according to 257 (59.6%) participants, and be able to do prayers (while kneeling) according to 215 (49.9%) participants. The most reported reasons that might prevent the participants from undergoing knee replacement surgery were the pain after surgery (59.4%), unavailability of surgeons (35.5%), and general complications from anesthesia (37.4%). Moreover, 188 (43.6%) participants thought that knee osteoarthritis is diagnosed using magnetic resonance imaging (MRI). Educational level was found to be associated with awareness score (*p* = .012). Conversely, gender, nationality, residence, and history of chronic disease were not found to be associated with awareness about total knee replacement (*p* = .548, .357, .734, and .639, respectively).

Conclusion: An average level of awareness and knowledge regarding total knee replacement was observed. Educational level was found to be significantly associated with this awareness.

## Introduction

Knee joint osteoarthritis is most common among older adults. Its prevalence is believed to be higher than 40% among people over 60 years [1]. In the later stages, the disorder needs to be treated surgically, through the method known as total knee arthroplasty (TKA) or total knee replacement. TKA restores knee activity and manages knee pain in osteoarthritic patients [2]. It is the most effective procedure to treat knee osteoarthritis. Moreover, it is also recommended for joints with dysplasia and malignancy [3,4]. Globally, the rate of use of TKA is increasing dramatically due to the increase in knee arthritis cases. It is expected that the primary TKA cases may increase to 85% by 2030 [5].

The first TKA was performed in 1986. The advancement in surgical techniques has enhanced its efficacy [6]. The National Joint Registry declared that more than 100,000 knees are replaced in the United Kingdom yearly [7]. In the United States, the National Institutes of Health (NIH) recommended some indications for the patients who required TKA: radiological proof of joint destruction, lasting and severe pain, and reduced life quality [8].

TKA is suggested at the end stage of knee osteoarthritis when the patient experiences severe pain and limited physical activity. The treatment of knees through TKA is more common among women than among men, with the most affected age range being 55-84 years. In the treatment, the knee is surgically removed and replaced by a prosthesis. However, some post-operative complications may arise, including venous thrombosis, inflammation, infection, osteolysis, and stiffness. Doctors can avoid these complications by suggesting the use of heparin, antibiotics, and knee movements post-operatively [8].

Apart from the above-mentioned complications, the patient's response and satisfaction are major factors in getting the relevant result for knee replacement. Patient response can affect pain and knee dysfunction, and reduced knee flexion can discourage the patient from giving a positive response to the treatment [9]. Some other factors affecting the success rate of TKA are age, weight, professional activity, height, and past trauma to the joint. Moreover, a high body mass index (BMI) is linked to greater use of knee replacement, more complications after the surgery, and limited implant durability [10,11].

The Middle East has also shown increasing cases of TKA in recent years due to its greater success rate. This has resulted in a higher survival rate. The adoption rate of this procedure has increased in various regions of the world [12]. In the Kingdom of Saudi Arabia (KSA), the osteoarthritis prevalence was reported at 13-30% in different areas [13]. However, the inconsistency in the adoption of TKA based on demographic features is evident; for example, women and older patients aged 75-79 years have greater TKA rates [12,14].

In the United States, older age and obesity will increase the TKA prevalence to 673% by 2030 [15]. Certain factors such as the knowledge level and social and cultural background of the patient also affect the patient's awareness and decision regarding TKA. In a US-based study, African Americans expressed greater willingness for TKA (80%) than white Americans (62%), due to a better understanding of the surgery and its positive after-effects in the former population [16].

Al-Omran reported satisfaction among the TKA patients in the KSA owing to better pain and knee functionality outcomes [17]. However, despite these positive outcomes, the rate of adopting this surgery is low in the KSA. In a study, about 67% of patients with osteoarthritis denied replacing their knees using TKA. The reasons to deny were misconceptions about the surgery and its implication, older age, and neglect of the severity of symptoms [18]. Such misconceptions affect the decision-making procedure of patients, and the lack of awareness prolongs their disease [19].

Therefore, it is necessary to increase the knowledge and awareness about TKA in patients with advanced osteoarthritis.

## Materials and methods

Objectives

• To study public awareness about total knee replacement among the Hail population.

• To explore the relationship between the level of awareness and different socio-demographic factors.

Methodology

Study Design

A cross-sectional study was carried out.

Study Area

This study was conducted in Hail, KSA, from August to October 2022.

Study Population

Participants were recruited from the general population.

Inclusion Criteria

Adult Hail city residents agreed to participate in the study, regardless of their nationality, who could read, and who had a social media account.

Exclusion Criteria

Non-Hail residents and those who had no social media account or refused to participate in the study.

Sample Size

The sample size was calculated using the Epi Info program (Centers for Disease Control and Prevention (CDC), Atlanta, US). The following parameters were utilized: 95% confidence interval, 5% margin of error, and the total population of Hail, KSA. The estimated sample size was 384, which was adjusted to 422 to compensate for the 10% non-response rate.

Data Collection Tools

The study was conducted using an online self-administered questionnaire created via Google Forms. The generated link was randomly shared on social media platforms (i.e., Facebook, WhatsApp, Telegram, and Twitter). The aim of the study was clearly explained in the interface.

A validated questionnaire was used based on previous studies. The questionnaire contained the socio-demographic characteristics of the participants, such as age group, sex, nationality, and residence. It also included questions about awareness of total knee replacement among the Hail population. A common grading method was used for each variable in this questionnaire as follows: 1 point was given to the correct option and 0 for the incorrect or neutral response.

Pilot Study

The questionnaire was pre-tested in a pilot study over a sample of 20 participants, but the results were not included in the study. Some modifications were done accordingly to ensure clarity of the questions.

Sampling Technique

A convenient non-probability sampling technique was employed to collect the data from the participants.

Data Analysis

Data were coded, entered, and analyzed using Statistical Product and Service Solutions (SPSS) (IBM SPSS Statistics for Windows, Version 23.0, Armonk, NY). Qualitative data were expressed in the form of numbers and percentages, and quantitative data in the form of mean and standard deviation. The Mann-Whitney test and Kruskal-Wallis test were used for continuous variables between two groups of data. A p-value below .05 was considered statistically significant.

Ethical Considerations

Due approval of the study was obtained from the Research Ethics Committee of Hail University. All participants were volunteers and asked to do their best. All data were kept confidential and used only for research purposes.

## Results

Participant characteristics

A total of 431 participants were included in the study. About 242 (56.15%) of the participants were males, and the rest 189 (43.9%) were females. Among these, 289 (67.1%) participants had studied up to university level, 86 (20%) had high school educational level, 35 (8.1%) were postgraduate, 17 (3.9%) had intermediate school level, and four (0.9%) were illiterate. The vast majority, that is, 397 (92.1%), were of Saudi Arabian nationality and 34 (7.9%) were of non-Saudi nationality. A total of 369 (85.6%) participants were residing in the city and 62 (14.4%) were in rural residences. Further, 79 (18.3%) participants had a history of chronic diseases, and the rest 352 (81.7%) had no chronic disease history. Table 1 presents the above-mentioned characteristics.

**Table 1:** Characteristics of the Study Participants (N=431)

Variable	Category	Frequency	% Percent
Gender	Male	242	56.1
	Female	189	43.9
Educational level	Illiterate	4	0.9
	Elementary school	0	0
	Intermediate school	17	3.9
	High school	86	20
	University	289	67.1
	Postgraduate	35	8.1
Nationality	Saudi	397	92.1
	Non-Saudi	34	7.9
Residence	City	369	85.6
	Rural	62	14.4
History of chronic disease	Yes	79	18.3
	No	352	81.7

Awareness about total knee replacement

Out of a total of 34 points, the average awareness score was 15 ± 6 (range: 5-33), with a mean percentage of awareness of 44.1%. The most reported causes of total knee replacement were overweight or obesity (n = 356; 82.6%), repetitive stress on the knee joint (n = 275; 63.8%), aging (n = 263; 61%), rheumatoid arthritis (n = 195; 45.2%), trauma to the knee joint (n = 180; 41.8%), inactivity (n = 162; 37.6%), wrong posture (n = 141; 32.7%), genetic factors (n = 78; 18.1%), bowing of legs (n = 70; 16.2%), and gender (n = 41; 9.5%). The most known symptom was pain, as reported by 369 (85.6%) participants; clicking sound during movements was reported by 279 (64.7%), swelling by 176 (40.8%), spasticity by 119 (27.6%), and bowing of the legs by 65 (27.6%) participants. Regarding the knowledge about the treatment, both physiotherapy and weight reduction were mentioned by 290 (67.3%), exercise by 244 (56.6%), surgical intervention by 177 (41.1%), medications by 121 (28.1%), and warm compressors by 112 participants.

**Figure 1:**
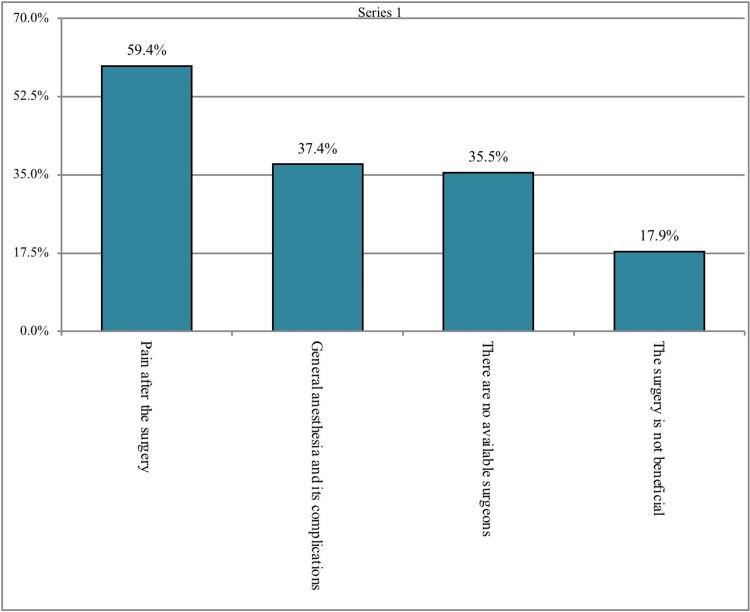
Reasons That Might Prevent Knee Replacement Surgery

**Figure 2:**
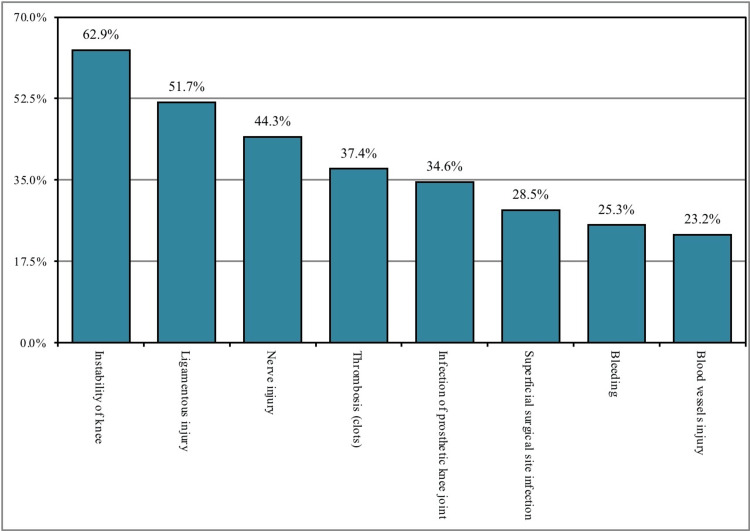
General Population's Perception Toward the Complications of Total Knee Replacement

A total of 357 (82.8%) participants thought the purpose of performing knee replacement surgery is pain relief, 257 (59.6%) of the participants mentioned that it to increase the walking quality, 215 (49.9%) of the participants mentioned that the purpose was to be able to do prayers, and 138 (32%) of the participants mentioned the main purpose is to go back to the sport. The reasons that might prevent the participants from performing knee replacement surgery were pain after surgery (n = 256; 59.4%), unavailability of surgeons (n = 153; 35.5%), general complications from anesthesia (n = 161; 37.4%), and consideration of surgery as unbeneficial (n = 77; 17.9%; Figure 1). Moreover, 343 (79.6%) participants thought that knee osteoarthritis can affect young people and 188 (43.6%) that it is diagnosed using magnetic resonance imaging (MRI), 97 (22.5%) that it is diagnosed with X-ray, 88 (20.4%) that it is diagnosed with computed tomography (CT) scan, and 58 (13.5%) that it is self-diagnosed. In addition, 374 (86.8%) participants thought that the surgical option is the best if non-surgical treatment fails. Regarding the side effects of total knee replacement, the following complications were reported: instability of the knee joint (n = 271; 62.9%), ligamentous injury (n = 223; 51.7%), nerve injury (n = 191; 44.3%), thrombosis (n = 161; 37.4%), prosthetic joint infection (n = 149; 34.6%), superficial surgical site infection (n = 123; 28.5%), bleeding (n = 109; 25.3%), and blood vessel injury (n = 100; 23.2%; Figure 2; Table 2).

**Table 2:** Awareness About Total Knee Replacement Among the Hail Population CT: computed tomography, MRI: magnetic resonance imaging

Variable		Frequency	% Percent
Cause	Overweight or obesity	356	82.6
	Repetitive stress on the knee joint	275	63.8
	Rheumatoid arthritis	195	45.2
	Aging	263	61
	Inactivity	162	37.6
	Wrong posture	141	32.7
	Bowing of the legs	70	16.2
	Trauma to the knee joint	180	41.8
	Gender	41	9.5
	Genetic factors	78	18.1
Symptom	Pain	369	85.6
	Swelling	176	40.8
	Spasticity	119	27.6
	Clicking sound during movement	279	64.7
	Bowing of the legs	65	15.1
Treatment	There is treatment	141	32.7
	Warm compressors	112	26
	Local injection of corticosteroids	163	37.8
	Physiotherapy	290	67.3
	Weight reduction	290	67.3
	Exercises	244	56.6
	Medications	121	28.1
	Surgical intervention	177	41.1
What do you think is the purpose of performing a knee replacement surgery?	Pain relief	357	82.8
	Enhancement in walking quality	257	59.6
	Ability to go back to sports	138	32
	Ability to pray while kneeling	215	49.9
Do you think that knee osteoarthritis can affect young people?	Yes	343	79.6
	No	27	6.3
	I don’t know	61	14.2
How do you think knee osteoarthritis is diagnosed?	Self-diagnosis	58	13.5
	Clinical examination	0	0
	X-ray	97	22.5
	CT-scan	88	20.4
	MRI	188	43.6
Do you think that surgical intervention is the best treatment if non‑surgical options didn’t work?	Yes	374	86.8
	No	57	13.2

Factors associated with awareness about total knee replacement

Educational level was found to be associated with awareness score (p = .012), as participants with university-level education tended to have higher awareness scores than others. Conversely, gender, nationality, residence, and history of chronic disease were not found to be associated with awareness about total knee replacement (p = .548, .357, .734, and .639, respectively) (Table 3).

**Table 3:** Association Between Socio-Demographic Characteristics and Awareness About Total Knee Replacement

Variable	Category	Awareness Score		p-value
		Mean	Standard Deviation	
Gender	Male	14.8	5.82	.548
	Female	15.2	6.29	
Educational level	Illiterate	16.8	2.06	.012
	Intermediate school	11.3	4.15	
	High school	14.1	5.25	
	University	15.2	6.28	
	Postgraduate	16.8	5.95	
Nationality	Saudi	14.9	6.06	.357
	Non-Saudi	15.8	5.74	
Residence	City	15.0	6.01	.734
	Rural	14.7	6.21	
History of chronic disease	Yes	14.5	5.44	.639
	No	15.1	6.16	

## Discussion

The lack of public awareness about total knee replacement is a significant issue as it might affect individuals' attitudes toward treatment. The assessment of this awareness will help determine the most efficient way of intervention in order to raise the level of awareness about particular conditions [20].

More than half (56.15) of the participants were males and the rest were females. The educational level for about two-thirds (67.1%) of the participants was university level. The vast majority (92.1%) were of Saudi Arabian nationality. The majority (85.6%) were city residents, and the rest of them were rural residents. Less than one-fifth (18.3%) had a history of chronic diseases, and the rest of them were with no chronic diseases.

The average awareness score was found to be 15 out of a total of 34 points. The mean percentage of participants with a good awareness level was 44.1%. These scores and percentages are lower than those reported in the congruent study (mean of 20.85 out of 35) carried out by Almaawi et al. (2022) [21].

The most reported causes of total knee replacements by the participants are overweight or obesity which was mentioned by the vast majority (82.6%) of the participants followed by (63.8%) who reported repetitive stress on the knee joint then (61%) mentioned aging and (45.2%) stated rheumatoid arthritis and others similar findings were reported in the parallel study which carried out by Momoli et al. (2017), wherein more participants mostly focused on mechanical injury as the cause of total knee replacement [22].

The most known symptom was pain, as reported by the vast majority (85.6%) of the participants followed by clicking sound during movements was reported by nearly two-thirds (64.7%) of the participants then swelling was mentioned (40.8%) of the participant, spasticity which reported by (27.6%) of the participants and others all these findings were agreed on by the study conducted by Heidari (2011), who reported pain, stiffness, and swelling as the main signs for knee osteoarthritis [23].

Concerning knowledge about treatment, both physiotherapy and weight reduction were mentioned by about two-thirds (67.3%) of the participants followed by exercise which was mentioned by more than half (56.6%) of the participants then surgical intervention as reported by (41.1%) of the participants and medications which mentioned less than one-third (28.1%) of the participants, weight reduction as a technique for reducing the stress on the knee joint was also demonstrated in the study that was conducted in Denmark [24].

The vast majority (82.8%) of the participants think the purpose of performing knee replacement surgery is pain relief whereas more than half (59.6%) of the participants mentioned that to increase the walking quality and nearly (49.9%) of the participants mentioned that the purpose was to be able to do prayers and others and this was found to be consistent with the findings of the study carried out by Baker et al. (2007) [25]. Regarding perception toward the complications of total knee replacement, nearly two-thirds (62.9%) mentioned instability of knee joint, half (51.7%) mentioned ligamentous injury followed by less than half (44.3%) mentioned nerve injury, about (37.4%) of the participants reported thrombosis and in about one-third (34.6%) of the participants mentioned infections of the prosthetic knee joint. The reasons that might prevent the participants from performing knee replacement surgery were pain after surgery as mentioned by more than half (59.4%) of the participants whereas more than one-third (35.5%) will not do it because there are no available surgeons and about (37.4%) of the participants will not do it due to the general complications from anesthesia and others all these perceived ideas were also demonstrated in the study conducted by Janse et al. (2004), who suggested that an effective doctor-patient relationship and proper counseling techniques would result in patient benefits [26]. Less than half (43.6%) of the participants think that knee osteoarthritis is diagnosed using MRI, one-fifth (22.5%) think that it is diagnosed with an x-ray, and (20.4%) think that diagnosis is with a CT scan this was also explained by Katz review (Katz et al., 2021) [27].

Furthermore, educational level was found to be associated with awareness score, but no such association was found with gender, nationality, and residence. These findings contradict the findings of Almaawi et al.'s (2022) study, which reported a significant association between gender and awareness level and no significant association between educational level and awareness [21].

Literature review

Knee osteoarthritis has been a prevalent disease due to increased age, obesity, and injury to the knee joint. At later stages of the disorder, patients with severe knee osteoarthritis have to undergo the surgical replacement of the knee joint. A study was conducted on 30 patients in the KSA to determine the radiological, clinical, and pathological aspects of knee osteoarthritis. This study included a significant number of women, individuals older than 60 years of age, and obese patients. Their pathological and radiological investigations showed high-grade lesions. According to the study, this finding will help patients with knee osteoarthritis across the world to decide on the TKA. The study was conducted on patients who had undergone knee replacement surgery, and post-operative improvements in their knees were examined through the knee society score, two-minute walk test, and timed up-and-go test. The patients involved had either degenerative or secondary osteoarthritis due to trauma. The results showed improved knee scores after surgery. Moreover, the timed up-and-go test also showed improvement in knee function with values ranging from 28.51 s to 18.18 s and 51.83 m to 85.72 m, respectively [28].

Another study in the same region was conducted to determine the factors associated with the prevailing osteoarthritis in older individuals. The results found growing age, female gender, high blood pressure, and high sugar levels as important factors. Therefore, patients with these factors should be monitored, as they are at high risk of end-stage knee osteoarthritis and may need TKA [13].

In another study, the attitude of Arab women toward total knee replacement was assessed by exploring their knee pain and limited movement. Discussions were conducted with five groups of patients. The collected data were transcribed and coded to determine the factors influential in deciding on TKA. The results enumerated the reason for the delay in opting for TKA, which included attempts to manage the disorder with drugs, fear of surgery, and poor knowledge about surgery. The study demonstrates the importance of providing knowledge to patients as part of the preoperative planning process. It also supports the doctor-patient relationship and encourages patients to cope with postoperative complications [29].

Al-Mohrej et al. (2017) investigated the knowledge and attitude of the public toward TKA in a study that involved 13 geographical areas, to cover the maximum population. They found about 30% awareness among the public, which was far less than the expected results. The factors affecting the awareness were age, financial status, and relatives with a history of TKA. This study's results can help provide the necessary information to improve the knowledge and awareness of TKA [30].

Youm et al. (2015) conducted valuable research to find out the effect of socioeconomic status (SES) of patients with knee osteoarthritis on their decision-making and choice of treatment. They reported that SES substantially impacts the efficiency of shared decision-making tools. Moreover, SES affects an individual's medical condition and their decision-making power about treatment. It was found that patients with private insurance are independent in their decision-making process. Further, a negative link was reported between education level and the decision of TKA [19].

## Conclusions

An average level of awareness and knowledge was found regarding total knee replacement, and educational level exhibited a significant association with public awareness. Therefore, efforts should be targeted toward raising the level of knowledge through effective mechanisms, including augmentation of the role of media and health education programs such as community events and social campaigns.
